# An emerging paradigm: a strength-based approach to exploring mental imagery

**DOI:** 10.3389/fnhum.2013.00104

**Published:** 2013-04-01

**Authors:** Tadhg E. MacIntyre, Aidan P. Moran, Christian Collet, Aymeric Guillot

**Affiliations:** ^1^Department of Physical Education and Sport Sciences, University of LimerickLimerick, Ireland; ^2^School of Psychology, University College DublinIreland; ^3^Centre de Recherche et d'Innovation sur le Sport, Université Claude Bernard Lyon 1France

**Keywords:** expertise, mental imagery, metacognition, motor cogniton, converging methods, mental practice, mental travel, mental rotation

## Abstract

Mental imagery, or the ability to simulate in the mind information that is not currently perceived by the senses, has attracted considerable research interest in psychology since the early 1970's. Within the past two decades, research in this field—as in cognitive psychology more generally—has been dominated by neuroscientific methods that typically involve comparisons between imagery performance of participants from clinical populations with those who exhibit apparently normal cognitive functioning. Although this approach has been valuable in identifying key neural substrates of visual imagery, it has been less successful in understanding the possible mechanisms underlying another simulation process, namely, motor imagery or the mental rehearsal of actions without engaging in the actual movements involved. In order to address this oversight, a “strength-based” approach has been postulated which is concerned with understanding those on the high ability end of the imagery performance spectrum. Guided by the expert performance approach and principles of ecological validity, converging methods have the potential to enable imagery researchers to investigate the neural “signature” of elite performers, for example. Therefore, the purpose of this paper is to explain the origin, nature, and implications of the strength-based approach to mental imagery. Following a brief explanation of the background to this latter approach, we highlight some important theoretical advances yielded by recent research on mental practice, mental travel, and meta-imagery processes in expert athletes and dancers. Next, we consider the methodological implications of using a strength-based approach to investigate imagery processes. The implications for the field of motor cognition are outlined and specific research questions, in dynamic imagery, imagery perspective, measurement, multi-sensory imagery, and metacognition that may benefit from this approach in the future are sketched briefly.

## Introduction

Since the classic study on mental rotations by Shepard and Metzler (1971) four decades ago, research on mental imagery, or “an internal representation that gives rise to the experience of perception in the absence of the appropriate sensory input” (Wraga and Kosslyn, 2002, p. 466), has flourished in cognitive psychology (e.g., Paivio et al., 1968), cognitive neuropsychology (e.g., Farah, 2000a), cognitive neuroscience (e.g., Kosslyn et al., 2006), and more recently, in the study of motor cognition (e.g., Jeannerod, 2001, 2006a). The common concern of researchers in these fields conveys the extent to which exploring mental imagery has become part of the cognitive science zeitgeist (Cornoldi and De Beni, 2012). Furthermore, *mental imagery* has also been of interest to those concerned with its application in the domains of skill acquisition (e.g., Sevdalis et al., 2013), rehabilitation sciences (e.g., Malouin and Richards, 2013) and professional expertise (e.g., Kozhevnikov and Blajenkova, 2013). What is it about mental imagery that has captured the interest of scientists across an array of disciplines for four decades? The answer to that question, as we shall see, is perhaps as complex as the construct of mental imagery itself.

Imagery, due to its ephemeral nature, has provided significant challenges for psychology since it first received formal research attention over a century ago (e.g., Galton, 1883). Chief among these challenges were the empirical question of how best to measure imagery given the limitations of the introspective methods (Kosslyn, 1980) and the theoretical question of whether the mental representation underlying imagery is propositional or analogical in nature (Block, 1983). Kosslyn et al. (2006) have provided a comprehensive proposal to resolve this latter issue. Despite such challenges, scientists have made significant progress in capturing the intricacies of mental imagery processes and applications by using new approaches (e.g., neuroscientific methods such as fMRI; Slotnick et al., 2005). Building on this need for new paradigms, the purpose of the present paper is to explore the possibility that a *strength-based* approach to imagery—one that focuses explicitly on recruiting participants at the high-ability end of the continuum of expertise—can augment conventional approaches to this construct. We argue that a strength-based approach may be valuable in illuminating both fundamental and applied questions (e.g., do expert athletes and/or dancers possess distinctive insights into meta-imagery processes in their domain?) which have so far evaded imagery researchers. In pursuit of this objective, the paper is organized as follows. In the first section, we shall review the strengths and weaknesses of conventional approaches to sampling that have dominated research on mental imagery for over a century. Next, we shall present an alternative approach to mental imagery research that we call the “strength-based” paradigm. The significant factors that led to the emergence of this paradigm will also be summarized. In the final section, we shall outline some potentially fruitful new directions in imagery research that can be addressed by supplementing traditional approaches with this strength-based paradigm.

## Key samples in mental imagery research

The study of imagery has profited greatly from neuroscientific methods (Behrmann, 2000). Given that it was a neglected topic for almost 50 years, its return from obscurity is remarkable (Kosslyn et al., 1995). Integral to the popularity of imagery as a legitimate scientific topic has been the increased strength in the inferences drawn from the research findings. These inferences are based on two aspects of the research process, the methodological tools and the approach to sampling (Table 1). The latter of these will be discussed in light of the converging methods approach of neuroscience (Kosslyn and Koenig, 1992).

**Table 1:** **Summary of traditional approaches to sampling in mental imagery research with human participants**.

**Samples**	**Paradigm**	**Method**	**Goal**	**Strengths**	**Limitations**	**Landmark study**
Experimenters were included in the sample of healthy subjects	Individual differences approach	Introspection	Understand phenomenology	Information appears to be easy to test	Highly subjective	Perky (1910) study of visual imagery and perception
Healthy subjects Large no. of subjects	Psychometric approach	Questionnaires	Focus on individual differences and sub-scale performance		Subjective Potential response bias (e.g., central tendency)	Marks (1973) Vividness of Visual Imagery Questionnaire (VVIQ)
Healthy subjects and typically right-handed (based on laterality tests) Large no. of subjects	Experimental approach	Response times (e.g., Mental scanning task)	Normalized data	Objective and easy to test large *N*	Trade-off between structure and process Some criticism of potential demand characteristics (Pylyshyn, 2003) and risk in use of sophisticated subjects	it took longer for subjects to consciously scan between image features that were relatively further apart than between those that appeared close together (Kosslyn et al., 1978)
	Cognitive neuropsychological approach	Correlational neural methods	Typically subtractive methods with normalized data combined with behavioral data			
Healthy subjects and clinical patients Systematic exclusion based on certain characteristics (e.g., imagery test performance)	nomothetic	EEG/ERP	Records changes in electrical potentials via electrodes	Relatively non-invasive measure of brain activity	Imprecise in resolution Only taps cortical changes Relatively expensive	EEG activity was measured in four tasks after sample was divided on the basis of VVIQ scores (Isaac and Marks, 1994)
Healthy subjects and clinical patients Small no. of subjects (<20)	nomothetic	PET/fMRI	Differences in the amounts of oxgyen consumed form basis for technique.	High spatial extent and resolution	Restricted movement for subject Highly expensive	Area 17 used in visual imagery and perception (Kosslyn et al., 1999)
Healthy subjects and clinical patients Small no. of subjects (<10)	nomothetic	TMS	Magnetic pulse can inhibit or facilitate function	Possible to target precise area	Low spatial extent Highly expensive	Used to inhibit imagery during rest condition prior to fMRI condition (Kosslyn et al., 1999)
Clinical patients 1-2 subjects with rare disorder	Neuropsychological case studies idiographic	Causal neural methods	Observation of patterns of dissociation and association	Ability to relate brain loci to functions	Uniqueness limits ability to generalize	Dissociation reported between mental imagery and object recognition in patient with brain damage (Behrmann et al., 1992)

Smith and Kosslyn (2007) describe the development of three main approaches in studying cognition: behavioral measures (e.g., latencies on computer-based tasks), correlational neural methods (e.g., fMRI), and causal neural methods (e.g., neuropsychological studies). Typical of this latter approach are comparisons between the performances of two groups on a task that requires imagery (i.e., behavioral data). The groups may comprise those who exhibit normal cognitive functioning or participants from clinical populations (Senior et al., 2006). Furthermore, the methods are often combined (i.e., behavioral data from normal and patient samples during brain imaging). Studying the deficits of patients with brain damage and impairments can establish that certain brain areas (e.g., by double dissociation) are at least partially responsible for a particular cognitive function. Neuropsychological evidence of both deficits following brain damage and brain activation patterns have been fundamental to the accumulating knowledge base of mental imagery (Kosslyn et al., 2006). Robust inferences can obviously be determined from monitoring changes in intact brain regions (Sarter et al., 1996), particularly when combined with theory-driven experimental paradigms (Kosslyn et al., 2006). Consequently, the neural-based studies with patients suffer from issues of generalizability but relying on converging evidence these limitations have largely been overcome.

From the above one could conclude that the cognitive neuroscience approach has encompassed participants across the performance spectrum, from those with deficits to those with high abilities. The nomothetic method has ultimately emphasized normative scores and even sophisticated statistical models are used to average date from neural imaging studies (Senior et al., 2006). Individual differences have not been central to the mental imagery research program in recent decades although as we shall see later in this paper, they provided an impetus for the strength-based approach. What is apparent from the aforementioned investigations is the paucity of research with participants at the high end of the continuum. Ability measures have been used widely to assess handedness (e.g., lateralization inventories) and they have been employed to discriminate good from poor imagers (e.g., Isaac and Marks, 1994). Indeed, highly skilled imagers may have been among the student samples in the classic imagery experiments of Kosslyn et al. (1978) and Shepard and Metzler (1971). The *deficit-based approach* which included both patients and healthy subjects may have overlooked potential insights, as we will propose later, from those who are highly skilled on imagery ability measures.

Nevertheless, the success of the prevailing paradigm has been the accumulation over four decades of substantive empirical evidence from case studies, behavioral data, and meta-analytic reviews (Kosslyn et al., 2006). Furthermore, evidence has emerged over the past two decades substantiating the neural basis of motor imagery (Munzert and Zentgraf, 2009; Munzert et al., 2009), olfactory imagery (Bensafi et al., 2003; Djordjevic et al., 2005), auditory imagery (Hubbard, 2010), gustatory imagery (Nitschke et al., 2006), spatial imagery (Kosslyn et al., 2007; Kozhevnikov and Blajenkova, 2013), and the role of emotions in imagery (Kosslyn, 2010).

However, some limitations in the key methods of cognitive neuroscience are also evident, and they may explain why our understanding of visual imagery is more comprehensive than that of other modalities (Moran et al., 2012). The scanning technologies to date (e.g., fMRI) restrict movement and thus limit maximal contractions during measurement. As a result, typically only discrete movements can be performed (e.g., small finger or foot movements; Pascual-Leone et al., 1995; Brown et al., 2005). Interestingly, transcranial magnetic stimulation (TMS) offers a different approach to studying action-one that is far more flexible as cortical functions can be disrupted even among normal controls (Walsh and Cowey, 2000; Stewart and Walsh, 2006). These methods may have obscured some aspects of imagery but we posit that in combination with the strength-based approach, they will ultimately illuminate the construct further.

## Antecedents of a “strength-based” approach

In many respects, the “strength-based” approach to understanding mental imagery is not a new phenomenon. In fact, we believe that it has three key antecedents—the individual differences approach to imagery (e.g., Galton, 1883), the idea of imagery as a cognitive simulation process (e.g., Jeannerod, 1994), and the emergence of a movement (known as “motor cognition”; see Jeannerod, 2006a) which is committed to the investigation of imagery-action relationships. As we shall explain below, we believe that these three antecedents have contributed certain key ideas which come together in the strength-based approach (Table 2). For example, the individual differences tradition led to the discovery that certain mental imagery processes are trainable. Similarly, the theory of “imagery as simulation” has helped researchers to postulate theoretical explanations for certain robust effects in imagery research (e.g., expertise effects in research on the phenomena of “mental practice” and “mental travel”; see explanation of terms below). Finally, the motor cognition movement has enabled theoretical bridges to be built between imagery research in cognitive sport psychology and that in neuroscience (see also Moran et al., 2012).

**Table 2:** **Antecedents to the strength-based approach in mental imagery research**.

**Issue**	**Source**	**Contribution**
Mental Practice (MP) effect	Vandell et al., 1943	Richardson (1967a,b) first narrative review of MP
Trainability of imagery	Shepard and Metzler, 1971	Subjects performed several thousand trials over 8–10 weeks
Mental travel effect	Decety et al., 1989	Congruence found between the duration of locomotion task and imagery
Converging methods approach	Kosslyn and Koenig, 1992	Kosslyn (1994) Neurally based computational model had superseded computational account (Kosslyn, 1980)
Role of deliberate practice in expertise	Ericsson et al., 1993	Expertise is domain-specific and is acquired through practice
Motor cognition approach	Jeannerod, 1994	Imagery is integral to motor preparation and action (Jeannerod, 2001, 2006a,b)
Individual differences in imagery ability	Kosslyn et al., 1998	MRT and rCBF study demonstrating two different ways to perform mental rotation, one that involves processes that execute movements and one that may not

### Individual differences

It has long-been known (e.g., since Galton, 1883) that people differ reliably from each other in their imagery experiences and skills. For instance, whereas some people can report intense visual and motor images, akin to actually “seeing” the experience and “feeling” the effort (e.g., running up a stairs), others report little detail in their recollection of imagery. This discovery of individual differences in imagery has led to several important if somewhat under-appreciated breakthroughs in our understanding of this construct. For example, consider Shepard and Metzler's (1971) study of mental rotations or people's ability to turn things over in their mind in order to answer questions about the spatial orientation of certain pairs of shapes. It is worth noting that the participants in this study were not naïve subjects but highly practiced individual who had performed several thousand trials over 8–10 weeks. Crucially, this study highlighted the fact that mental rotation processes were not only measurable but *trainable* too. Interestingly, the extensive practice trials sparked follow-up research on the role of *practice* in mental rotation effects (Steiger and Yuille, 1983). Results showed that mental rotation effects were robust—regardless of the amount of training received by participants (Leone et al., 1993). Another notable finding from imagery studies in this era concerns Kosslyn's (1994) report that Jacky Metzler (co-author with Roger Shepard) had commented that some subjects had experienced what appeared to be “kinesthetic” sensations during imagery. It now seems that the mental rotations task, which was originally assumed to be visuo-spatial in nature, may actually involve the *motor* system. This discovery of motor involvement in visuospatial imagery could account for the early findings in neuroimaging studies of mental rotation that reported multiple brain areas activated during the task (Kosslyn et al., 2001a). Indeed, Kosslyn had argued that “visual mental images are transformed in part via motor processes” (1994, p. 345). A key question arose for researchers: do different strategies influence performance on mental rotations ability? In 1998, an rCBF technique was employed while subjects mentally rotated either hands or the original 3-D block objects (Kosslyn et al., 1998). The results highlighted that two mechanisms could be applied—“one mechanism that recruits processes that prepare motor movements and another that does not” (Kosslyn et al., 1998, p. 151). Findings on the neural basis of mental rotation, while dependent on the type of stimuli (animate vs. inanimate), may be *contaminated* by some subjects using a motor-based strategy. In a follow-up study, subjects either imagined rotating 3-D block isomers by hand or by a motor and the neural evidence demonstrated differential activation of the motor and visual cortex, respectively (Kosslyn et al., 2001b).

Another consequence of the individual differences movement was the range of expert samples used (e.g., pilots; Dror et al., 1993) and the range of methods expanded to explore in an ecological way the application of mental imagery in everyday life. For example, a weeklong diary study found that students reported using imagery for a range of purposes including problem solving (e.g., navigation) and mental practice (Kosslyn et al., 1990).

### Imagery and simulation

The idea of imagery as a cognitive simulation technique may be traced back to William James' (1890) ideomotor principle (Jeannerod, 2006b). Since then, two different imagery effects have come to the attention of researchers—firstly, “mental practice,” and secondly, “mental travel.”

Firstly, mental practice, or “the systematic use of mental imagery to rehearse an action in the minds eye without engaging the actual physical movements involved” (Moran, 2012, p. 349), has been subjected to formal study since the 1940's (Vandell et al., 1943). In the intervening decades several hundred studies have investigated the mental practice effect, which based on meta-analytic research has a significant positive effect on the performance of motor skills (Driskell et al., 1994). A number of moderating variables were reported including the type of task and experience level. To explain, experienced athletes appear to benefit more from mental practice than do novices. This finding suggests a possible constraint on the efficacy of mental practice for novice learners. Specifically, as Driskell et al. (1994) proposed, that for “novices, who have not formed an approximation of the skill, the symbolic rehearsal provided by mental practice may not be sufficient to guide performance” (p. 489). One explanation for this expertise effect within mental practice is that the enhanced imagery abilities of experts may enable them to develop greater implicit knowledge of the spatial and kinesthetic requirements of the tasks than is possible for relative novices. Alternatively, experts may simply possess greater meta-cognitive knowledge of how to *employ* imagery effectively for skill improvement as compared with novices.

Since the early 1990's researchers have continued to show how mental practice can improve sport skills (e.g., flip-turn in swimming; Casby and Moran, 1998), surgical skills (Arora et al., 2011), and can accelerate the process of neurorehabilitation (McEwan et al., 2009). However, many questions remain unanswered. For example, what are the exact psychological mechanisms underlying mental practice effects (Kosslyn and Moulton, 2009)? How best does one apply imagery optimally in motor skill rehearsal (Weinberg, 2008)? And how do elite athletes employ mental practice in field settings (Moran, 2012)? Nevertheless, the mental practice literature highlighted the clever application of imagery among elite sport participants (Morris et al., 2005). And furthermore, the awareness of the mental practice effect created a common interest for sport psychologists and cognitive neuroscientists alike. While the former were primarily concerned with performance as the dependent variable, the latter were interested in the neural basis. In fact, researchers within the field of sport psychology have called for models of mental imagery in sport to be grounded in the neuroscientific literature and attempts at such theorizing have been tried (Holmes and Collins, 2001; Guillot and Collet, 2008; Wakefield et al., 2013).

The second imagery effect that has been reported in the literature is the mental travel effect, which is concerned with the relationship between the duration of a simulated movement (i.e., through imagery) and its executed counterpart (Decety et al., 1989). For example, in the early study by Decety et al. (1989), participants had to perform a blindfold walk and then mentally simulate the walk. Congruence between the duration for the tasks in each condition was reported in this study. Since then a robust mental travel effect has consistently been found while both underestimation and overestimation may occur in certain conditions, the temporal coupling of simulated and executed action is strongly influenced by expertise. Reviews have indicated that elite performers in sport are highly proficient at this skill (Guillot and Collet, 2005; Guillot et al., 2012a,b). For example, strong correlations have been reported between the time taken to rehearse completing a canoe-slalom course and the motor execution time (Moran and MacIntyre, 1998). Conversely, patients with developmental movement disorder (e.g., Gabbard et al., 2012), and a variety of neurological disorders of the motor system have been demonstrated to perform poorly on tests comparing the duration of simulated and executed movements (Guillot and Collet, 2005).

### Motor cognition

As a consequence of the robust findings on imagery effects, motor imagery rose to prominence at the interface between cognitive neuroscience and sport psychology (Moran et al., 2012). Since Jeannerod's landmark paper in 1994, the implications of motor imagery as a window into the representation of action have been acknowledged by numerous researchers (e.g., Guillot and Collet, 2005; Smith and Kosslyn, 2007; Bläsing et al., 2012). The adoption of this new umbrella term, *motor cognition*, may now ensure that the study of action is within the realm of cognitive neuroscience after a degree of neglect by researchers within psychology (Rosenbaum, 2005). By implication the domains of dance (Bläsing et al., 2010), sport (Kosslyn and Moulton, 2009) have all, as a consequence, evolved as natural laboratories for the study of motor cognition. Why the neglect of motor control by psychology? Rosenbaum (2005) argues that “motor control has had the status of Cinderella in psychological research” (p. 308). Among the reasons for the failure of psychology to engage in attempts to understand aspects of motor control were the *“too hard to study hypothesis.”* Undoubtedly the complexity of psychological aspects of motor control was a barrier to research, but the *raison d'être* may be that the methodological barriers hindered its exploration. It seems plausible to propose that motor imagery has only become a topic of study since researchers have had the methodological tools to explore it (i.e., through mental kinematics). The aforementioned factors, allied with the expertise paradigm, based on the theory of deliberate practice (Ericsson et al., 1993; Ericsson, 2009) provides the impetus for the strength-based approach.

## The strength-based approach to studying mental imagery

The “strength-based” approach provides an extension of the prevailing paradigm of cognitive neuroscience, which has focused primarily upon investigations with both healthy subjects and patient samples. Instead, the emphasis is on those at the high ability end of the continuum. Furthermore, it posits that an expertise paradigm should be employed, to ensure that a comprehensive rationale is provided for the selection of participants (i.e., they meet multiple criteria to establish their level of performance). To tap this expertise efficiently, it is proposed that the converging methods approach is applied and moreover, that ecological validity is considered in the experimental design (Table 3).

**Table 3:** **Proposed assumptions of the strength-based approach**.

**Assumption**	**Implications**
Expertise approach (Ericsson et al., 1993)	• Use the dimension of expertise to choose samples
	• Employ multiple criteria to establish level of expertise
	• Explore expertise across domains relevant to mental imagery processes
Ecological validity (Neisser, 1976, 1978)	• To preserve the domain-specific expertise elements of ecological validity should be included in the study design
	• This should occur across the different dimensions-nature of the setting, stimuli, and response set
Converging methods (Kosslyn and Koenig, 1992)	• This approach should be employed to explore the interaction between abilities, the brain and computation
Theory-based approach	• Research questions should be guided by theory
	• Functional-equivalence and structural equivalent accounts of mental imagery provide a road map for research questions

Two key issues integral to the application of the strength-based approach are now highlighted. These are quantifying expertise and applying the principle of ecological validity. Subsequently, recent examples of this approach in music, sport, and dance samples are discussed. And finally, we highlight where we foresee the “strength-based” approach can illuminate the interaction between cognition and action (motor cogntion), the integration of multi-sensory information in perception and simulation, and the role of conscious thought and knowledge in imagery (metacognition).

### Expertise paradigm

Expert performance is defined as consistently superior performance on a specified set of representative tasks for the domain that can be administered to any subject (Ericsson, 2009). Ericsson and Smith (1991) proposed the expert performance approach to understand the critical mechanisms underlying expertise. Thus, the “strength-based” approach advances the prevailing research paradigms by explicitly exploring experts to enhance our understanding of mental imagery. As we have noted, this had been done previously but it occurred in an implicit fashion. For instance, the training in the original mental rotation study hinted at the development of expertise (Shepard and Metzler, 1971). Only recently have experts been targeted consistently as samples by cognitive neuroscientists (Table 4). Notable exceptions include the use of expert samples (i.e., artists) by Blajenkova et al. (2006) in their validation of the *Object-Spatial Imagery Questionnaire*. Another example of the implicit expertise approach was the inclusion of US air force pilots in a study by Dror et al. (1993) on their visual-spatial ability. However, in that case, we would assert that their expertise should be quantifiable across multiple criteria including their memory, metacognitive skills, and not simply the number of flying hours accrued.

**Table 4:** **Recent studies using neuroimaging methods in mental imagery with music, sport, and dance samples**.

**Question**	**Method**	**Authors**	**Sample**	**Findings**
An attempt to define any association between activated brain areas and golf skill	fMRI of imagery of golf swing	Ross et al., 2003	6 golfers from novice to elite level	Decreased brain activation occurred with increased golf skill level for the SMA and cerebellum with little activation of basal ganglia
Investigation of the cortical network which mediates music performance compared to music imagery	fMRI	Meister et al., 2004	12 music academy students	Activations of premotor areas and precuneus were found in both conditions, contralateral M1 and posterior parietal cortex were active during performance only
Comparison of neural networks of expert and novice golfers during simulation of pre-shot routine	fMRI of imagined pre-shot routine	Milton et al., 2007	6 expert and 7 novice golfers	Extensive practice leads experts to develop a focused and efficient organization of task-related neural networks, whereas novices have difficulty filtering out irrelevant information
To investigate differences in brain activity between groups and to effect of the use of internal vs. external perspective	fMRI of motor imagery of a high jump	Olsson et al., 2008	High-jumpers (12 elite and 12 novices)	Imagery training reduces the activity in parietal cortex suggesting that imagery is performed more automatic and results in a more efficient motor representation more easily accessed during motor performance
Role of experience in facilitating corticospinal representations of actions	TMS of familiar and unfamiliar skills	Fourkas et al., 2008	3 expert tennis players	Subjective reports indicated that only in the tennis imagery condition did experts differ from novices in the ability to form proprioceptive images
To investigate multi-modal musical imagery performed by expert pianist	fMRI during imagery and simulated motor performance of a memorized extract	Davidson-Kelly et al., 2011	42-year-old expert pianist	Pattern of activation for performed and imagined piano music was similar, with the motor system of the brain showing similar activation during both conditions (except for M1)
Study of dynamic neurofunctional changes induced by a physical training	fMRI of golf putts in longitudinal study over 40 h training	Bezzola et al., 2012	11 novice golfers and age-matched controls	Training induces functional neuroplasticity and skill improvement is associated with a modified activation pattern

The challenge for researchers, therefore, is to quantify precisely what constitutes an expert. Standardized ranking systems within domains like chess make this possible (Saarliluoma et al., 2004) but in sport, dance, and music, it is more difficult to have a standard metric. Typically researchers in sport have defined an expertise as a function of competitive level (e.g., novice, collegiate, elite, professional). This simple rubric, with elite being denoted as those competing at the highest possible level (Van den Auweele et al., 1993) is vague and may not adequately reflect the nature of the expertise. For example, is an elite athlete equivalent to a chess Grandmaster? Consequently, there is a need to fractionate experts from one another in terms of expertise. In domains such as music, dance, and sport “performance can be publically observed and even objectively measured in open competition and public performances” (Ericsson, 2009, p. 18). And moreover, given that expertise is distinguishable according to criteria, including metacognition (Ericsson, 2009), the precise performance across a matrix of measures of expertise should be explored (e.g., declarative knowledge, predictive ability).

Furthermore, comparisons across expert groups should be applied rather than simply exploring expert-novice differences. Experts and novices can easily be discriminated from one another in many domains on such an array of variables that the comparison can be meaningless. Instead the emphasis should be on inclusion criteria that target participants on the expertise spectrum based upon the research question. To explain, while the studies listed in Table 4 have samples that reflect a range of abilities, it is evident that purposive sampling was applied. For example, to understand learning a novice sample were used (Bezzola et al., 2012) and on the other hand, a spectrum of abilities were represented in Ross et al.'s (2003) investigation of links between activated brain areas and golf skill.

The primacy of the expertise approach within the “strength-based” approach supplements the prevailing approach with the intention of exploring the role of expertise in mental imagery processes. Experts can be recruited, for instance, on the basis of their special imagery abilities or their sport, or professional activity expertise. Moreover, choosing appropriate activities from which to recruit samples should be based upon the cognitive task-demands of the actions, rather than an *ad-hoc* decision. One paradox is that clinical patients may develop specialized abilities in order to cope with the demands of their deficits. One such example is the case of IW, a patient with peripheral neuropathy, who has shown diminished motor imagery ability but enhanced visual imagery ability relative to controls (ter Horst et al., 2012). The inclusion of expertise as a variable in mental imagery research can shed light on both the processes underlying imagery and its potential application.

### Ecological validity

The expert performance approach of Ericsson and Smith (1991) proposes that field or laboratory tasks are designed in order to retain a high level of ecological validity. As Saarliluoma et al. (2004) states “it is important to vary the way basic concepts such as mental imagery are operationalized to avoid the metascientific *Ebbinghaus effect*” (p. 753–754). In other words, complex and dynamic tasks in which imagery processes are important should be subject to scientific scrutiny or aspects of the underlying processes may be overlooked. One persistent criticism of imagery research within the sport context is the use of tasks lacking ecological validity (Morris et al., 2005; Moran, 2012). This challenge applies to perhaps a greater degree within neuroscience where simple laboratory tasks (usually involving constrained movements of the fingers) that are chosen not only for their amenability to computational modeling but also for the ease with which they can be mastered after a relatively small amount of practice (Nielsen and Cohen, 2008; Yarrow et al., 2009). Ecological validity is a necessary component of studies that are targeting expertise as if we are to determine their abilities within a converging methods approach, some transfer of skill and process should occur (Moran, 2012). The dimensions of ecological validity include the nature of the setting, the stimuli, and the response (Schmuckler, 2001). These interlinked dimensions can be considered within the converging methods approach that is integral to the “strength-based” approach. For example, the diary study methodology conducted by Kosslyn et al. (1990) could be utilized to evaluate experts' imagery use over time (e.g., professional dancers). This approach offers researchers a naturalistic laboratory for investigating imagery and action (Moran, 2009).

## New approaches to old questions

Borrowing from Boring (1957), it may be argued that imagery research has a long past but only a short history and that some enduring questions in this field remain unanswered. As we have noted, perennial issues around imagery measurement, theory, and function have been central to enquiry for over a century (Roekelein, 2004). For instance, Jeannerod (2006b) states that the “mental conception of action” or the “motor idea,” “to account for the role of memory images or remote impressions in shaping an action” has been with us since the time of William James (p. 360). The key issue is how the prevailing paradigm of cognitive neuroscience has shifted in recent decades. We have seen how the field of cognitive psychology has been reconstituted since the 1990s with an increasing emphasis on neural implementation (Smith and Kosslyn, 2007; Anderson, 2010). Moreover, the key topics that delineated cognitive psychology in the early textbooks have been extended to include neuroscience methods, emotion, and action (Smith and Kosslyn, 2007). Furthermore, Martha Farah has stated that the present paradigm of cognitive neuroscience “is far from having outlived its usefulness … and I'd like to see it continue to move towards the edge of our understanding” (2000b, p. 362).

The evidence presented heretofore, on the growth of the “strength-based” approach, conveys how it has augmented the study of abilities within the cognitive neuroscience triangle (Kosslyn, 1994). The expansion of the “strength-based” approach within mental imagery research opens up new modes of enquiry for mental imagery and perception (e.g., Tartaglia et al., 2009) and specifically in our understanding of action processes. Motor cognition research has the potential to shed light on imagery processes, the representation of action, and the role of imagery processes in experts.

### Motor cognition

Three key questions within the field of motor cognition can be understood by applying the “strength-based” approach. One issue surrounds the role of action coupled with motor imagery, what has been termed dynamic imagery (MacIntyre and Moran, 2010). Motor imagery, by definition, occurs in the absence of any overt movement or motor output (Guillot and Collet, 2010). However, on the basis that athletes often engage in movements while engaging in imagery, sport psychologists have recommended that performers apply this in their imagery practice (Holmes and Collins, 2001) and moreover, they have amended their definition of mental imagery to include possible motor output (Morris et al., 2005). Researchers had noted that athletes engaged in either synchronous movements (e.g., simulating the task) or asynchronous movements (e.g., other movements) during imagery (MacIntyre and Moran, 2010). The role of these quasi-movements (Nikulin et al., 2008) has yet to be rigorously evaluated. Preliminary evidence suggests that athletes find this beneficial (MacIntyre and Moran, 2010) but to date research has not investigated the complex issue of coupling action and motor imagery by athletes (for an exception see Guillot et al., 2013). Evidence from other samples suggests that this is a topic worthy of further research. For example, Ionta et al. (2012) reported that variations in the hand position of participants' during mental rotations tasks influenced the latencies for congruent stimuli. They concluded that sensory-motor and postural information coming from the body might influence mental rotation of body parts according to specific, somatotopic rules. These preliminary findings were congruent with the *body-specificity hypothesis* that claims that body-specific patterns of motor experience shape the way we think (Casasanto, 2011). Furthermore, future findings from this line of enquiry may have ramifications for the recent accounts of embodied cognition (Borghi and Cimatti, 2010; Gallese and Sinigaglia, 2012). One confound that has been noted in research on the action-motor imagery coupling is the imagery perspective (the viewpoint adopted during visual imagery). This has resulted in debate in both sport psychology and cognitive neuroscience. Moran (2012) noted that the complexity of agency, visual perspective, and confounds with kinesthetic or motor imagery were reflected in the findings emanating from sport psychology. Researchers had developed sophisticated methodologies to attempt to control and measure the visual perspective adopted during testing. The “strength-based” approach is one possible route to understanding this topic further. A recent special issue of the *Journal of Mental Imagery* on whether the internal viewpoint is a default hypothesis is testament to the continuing interest in this topic (Morris and Spittle, 2012). The issue of visual perspective in mental imagery, because of the conflation with both visual imagery and motor imagery in the past, has been noted as a topic that necessitates further research (Madan and Singhal, 2012).

A second question that relates to motor cognition is the process of multi-sensory integration, which is an area of current debate (Foxe and Molholm, 2009). More specifically, the relative contribution of different senses in the simulation of action is of direct concern to neuroscientists (Lacey and Lawson, 2013). Recently, this topic has received increased attention because of concerns with the traditional approach in the understanding and application of multi-sensory imagery. For example, within sport psychology, it was traditionally assumed that a multi-modal approach was optimal (Morris et al., 2005). However, this has recently been questioned on the basis that it may be more important to only imagine the pertinent senses (Holmes and Collins, 2001; Weinberg, 2008; MacIntyre and Moran, 2010). Initial evidence, from fMRI studies (Ross et al., 2003), dual-task studies (Smyth and Waller, 1998), and qualitative accounts of how elite performers employ imagery (Munroe et al., 2000; MacIntyre and Moran, 2007a,b) suggests that the simplistic multi-sensory application of imagery merits further investigation. Interestingly, this topic would be of interest across the domains of music, sport, and dance that have been referred to in this paper. From another perspective, the importance of different senses underlying the reported imagery effects has yet to be fully ascertained and both inhibitory processes during imagery and consolidation effects are only beginning to be explored (e.g., Guillot et al., 2012a,b).

Fourthly, as discussed in the introduction, measuring imagery ability has been an issue of controversy for the field since Galton's early attempts at quantification (Galton, 1883). The question “why do people differ so much in their imagery abilities” (Kosslyn et al., 2001a, p. 641) is still pertinent today. The influence of implicit or explicit expertise on mental rotations findings, in mental travel research, and in mental practice studies has been established (Guillot and Collet, 2005). And moreover, a trend in imagery research has been the expansion of the imagery ability from one unitary construct to a number of distinct abilities which reflect different neural processes (e.g., dorsal vs. ventral stream; Blajenkova et al., 2006). What is less clear is how imagery abilities are developed and what is the precise role of these imagery abilities in moderating imagery effects (Madan and Singhal, 2012). One alternative to the plethora of pencil and paper imagery tests employed in sport (e.g., Williams and Cumming, 2011) or neuroscience settings (e.g., McAvinue and Robertson, 2007) and is to employ a compound measure, the motor imagery index, which combines psychometric, behavioral (e.g., mental travel), and psychophysiological measures (Collet et al., 2011). It is vital that we are able to quantify imagery abilities if we are to match participants for competence or if we wish to evaluate the trainability of imagery abilities. Recent research has explored the trainability of imagery vividness using robust measures and interestingly, the only reported changes were in the metacognitive understanding of their imagery (Rademaker and Pearson, 2012).

### Meta-imagery

Theoretically, a potentially valuable new route for imagery researchers in cognitive neuroscience concerns the investigation of the neglected topic of “meta-imagery processes”—“their beliefs about the nature and regulation of their own imagery skills” (Moran, 2002, p. 415). Research in the expertise literature suggests that meta-cognition, people's insight into, and control over their own mental processes, is a factor that differentiates novices from experts (Moran, 2012). Interest in this topic surprisingly emerged from a survey by Denis and Carfantan (1985) who surveyed undergraduate students on their knowledge about imagery research findings in psychology. The motive for their study was to quantify the participants' *tacit knowledge* of imagery effects (Denis, 2012). In order to assess the level of *tacit knowledge* among experimental subjects they were asked to predict the outcomes of various imagery experiments that were described but not formally named (e.g., is memory for pictures better than memory for words?). The findings indicated that although the majority of participants predicted correctly that imagery would have beneficial effects on learning and reasoning, few subjects were able to predict accurately the results of mental rotation experiments (whereby more time is required to accomplish greater amounts of rotation of images) or mental scanning studies (whereby longer distances between points in an image take longer to scan than shorter distances). Furthermore, a majority of participants rejected the idea that mental imagery could enhance the performance of motor skills (the mental practice effect). This latter finding led Denis and Carfantan (1985) to conclude “how counterintuitive the idea is that motor skills may be affected by purely mental practice” (p. 56). The naïve responses of the participants in this study are in stark contrast to the evidence that has recently emerged from sporting samples. Researchers have asked athletes and dancers to indicate why, where, how, what, and when they use mental imagery processes (e.g., Nordin and Cumming, 2005; MacIntyre and Moran, 2007a,b). Athletes' responses from both interviews and surveys demonstrated a comprehensive knowledge of the multi-model potential of imagery, their awareness of both mental practice and mental travel effect, and highlighted the sophisticated nature of their understanding of imagery (MacIntyre and Moran, 2010).

In 2002, Moran suggested that it would be interesting to discover if top athletes have greater insight into and control over their use of imagery compared with their less successful counterparts. To date the preponderance of the evidence favors an expertise effect for meta-imagery. In fact, a model of meta-imagery in action suggests that there are three components-knowledge, monitoring and control, which opens up possibilities of developing a test of meta-imagery (MacIntyre and Moran, 2010). Furthermore, contemporary evidence from cognitive psychology supports the role of meta-cognitive knowledge of imagery ability and relates it to our ability to judge individual episodes of imagery (Pearson et al., 2011). The voluntary nature of imagery and the role of conscious awareness during imagery tasks make it amenable to introspection (Pearson et al., 2008), ironically the method that was central to the demise of the scientific study of imagery, a century ago (see Block, 1983). While in the past the study of metacognition has targeted intellectual skills “if intellectual and perceptual-motor skills rely on similar mechanisms, one would expect metacognition to apply to the guidance of perceptual-motor skills, just as it does to the guidance of intellectual skills” (Augustyn and Rosenbaum, 2005, p. 911). Consequently, armed with a comprehensive roadmap of imagery processes and an increased understanding of action, the study of meta-imagery could provide a back door into the typically impenetrable realm of sensory perception (Pearson et al., 2011).

## Some observations on the strength-based approach within cognitive neuroscience

In this paper, we have argued that a strength-based approach to mental imagery can augment rather than replace the traditional approach to this construct. After all, this latter approach has been highly successful in, for example, illuminating both visual imagery and visual cognition (Kosslyn et al., 2006). More recently, this latter approach has led to advances in our understanding of the overlap between visual perception and imagery in scanning tasks (Borst and Kosslyn, 2008). Based on such progress, there is an imperative for researchers to maintain the traditional approach in order to answer key questions. For example, the use of randomized controlled trials to explore the role of motor imagery in patients with sub-acute neglect (Welfringer et al., 2011) and with stroke victims (Ietswaart et al., 2011) are essential for the validation of imagery interventions. Similarly, the role of mental imagery among clinical patient samples (Pearson et al., 2012) and stroke victims (Confalonieri et al., 2012) requires continued investigation. Unsurprisingly, studies of patients will, in all likelihood, continue to inform our appreciation of the deficits, challenges, and recovery strategies of those with specific with rare disorders (ter Horst et al., 2012). This again raises aforementioned paradox of expertise among patient samples. A recent study with a Paralympic athlete led to the conclusion that only tasks that we have physical experience of recruit the motor system (Olson and Nyberg, 2011).

Despite the success of traditional approaches to imagery, however, a strength-based approach may contribute in the development and refinement of imagery inventories, as implicitly employed in the case of the *Object-Spatial Imagery Questionnaire* (Blajenkova et al., 2006). Similarly, investigations of the neural processes underlying imagery effects in expert samples will help to elucidate both applied and theoretical aspects of mental imagery. However, the new approach advocated in this paper has its limits too. For example, analyzing the imagery skills of motor experts in an atheoretical manner will not advance conceptual understanding and may provide spurious findings. Instead, a rigorous, theory-driven approach with converging methods is required for strength-based approaches to yield benefits to imagery researchers. As anticipated by Kosslyn et al. (2002) over a decade ago, “individual differences can actually help to reveal the nature of underlying mechanisms (p. 342).”

## Conclusions

Cognitive neuroscience has made impressive progress in the illumination of the nature, function, and neural basis of mental imagery (Kosslyn, 2010). Nevertheless, certain aspects of this construct (e.g., its relationship to skilled performance) have been relatively neglected by mainstream imagery researchers. In this article, certain significant trends—antecedents of a strength-based approach to imagery—that may be detected over more than a century of imagery research have been highlighted. These trends provide signposts for at least four potentially fruitful new avenues of inquiry. Firstly, imagery research continues to evolve from an excessively narrow focus on the visual sense (e.g., Galton, 1883; Perky, 1910) to a modern concern with investigation of the true multi-sensory character of this construct. Next, theoretical understanding of mental imagery has deepened with increasing awareness of the multi-dimensional nature of this construct. Thirdly, major methodological advances, both in technology and sampling, have illuminated both fundamental and applied questions. Finally, the preliminary application of the “strength-based” approach has been shown to be useful in enriching our understanding of the neural basis of expert performance (Ross et al., 2003; Milton et al., 2007; Olsson et al., 2008) and the “clever application” of imagery in sport (Moran et al., 2012), dance (Bläsing et al., 2012), and music contexts (Meister et al., 2004). It is noteworthy that the interest in exploring the naturalistic expertise in imagery (e.g., dance) with neuroscientific methods stems is bi-directional. For example, “neuroscientists have turned to dancers as a valuable human resource in possession of a rich skill set … to address issues of how the brain coordinates perception with action” (Cross, 2010, p. 197). The implications of augmenting existing paradigms with the “strength-based” approach will be most obvious in the domain of motor cognition where issues of dynamic imagery, visual imagery perspective, and multi-modal integration can be explored. Also, the area of metacognition research is a rapidly growing field and meta-imagery as a topic is evolving. Meta-imagery research is a topic than can benefit from the “strength-based” approach. In summary, through the lens of motor cognition, further exploration of the construct of mental imagery will ensure that the topic of will remain part of the cognitive neuroscience lexicon for many decades to come.

### Conflict of interest statement

The authors declare that the research was conducted in the absence of any commercial or financial relationships that could be construed as a potential conflict of interest.

## References

[B1] Anderson J. R. (2010). Cognitive Psychology and its Implications. New York, NY: Worth Publishers

[B2] Arora S. Aggarwal R. Sirimanna P. Moran A. Grantcharov T. Kneebone R. (2011). Mental practice enhances surgical technical skills: a randomized controlled study. Ann. Surg. 253, 265–270 10.1097/SLA.0b013e318207a789 21245669

[B3] Augustyn J. S. Rosenbaum D. A. (2005). Metacognitive control of action: preparation for aiming reflects knowledge of Fitts' Law. Psychon. Bull. Rev. 12, 911–916 16524010 10.3758/bf03196785

[B4] Behrmann M. (2000). The mind's eye mapped onto the brain's matter. Curr. Dir. Psychol. Sci. 9, 50–54

[B4a] Behrmann M. Winocur G. Moscovitch M. (1992). Dissociation between mental imagery and object recognition in a brain-damaged patient. Nature 359, 636–637 10.1038/359636a0 1406994

[B5] Bensafi M. Porter J. Pouliot S. Mainland J. Johnson B. Zelano C. (2003). Olfactory activity during imagery mimics that during perception. Nat. Neurosci. 6, 1142–1144 10.1038/nn1145 14566343

[B6] Bezzola L. Merillat S. Jancke L. (2012). The effect of leisure activity golf practice on motor imagery: an fMRI study in middle adulthood. Front. Hum. Neurosci. 6:67 10.3389/fnhum.2012.00067 22479243 PMC3315099

[B8] Blajenkova O. Kozhevnikov M. Motes M. A. (2006). Object-spatial imagery: a new self-report imagery questionnaire. Appl. Cogn. Psychol. 20, 239–263

[B9] Bläsing B. E. Calvo-Merino B. Cross E. S. Jola C. Honisch J. Stevens C. J. (2012). Neurocognitive control in dance perception and performance. Acta Psychol. 139, 300–308 10.1016/j.actpsy.2011.12.005 22305351

[B10] Bläsing B. Puttke M. Schack T. (2010). The neurocognition of dance. Introduction, in The Neurocognition of Dance: Mind, Movement and Motor Skills, eds Bläsing B. Puttke M. Schack T. (Hove, East Sussex: Psychology Press), 1–8

[B11] Block N. (1983). Mental pictures and cognitive science. Philos. Rev. 92, 499–541

[B12] Borghi A. M. Cimatti F. (2010). Embodied cognition and beyond: acting and sensing the body. Neuropsychologia 48, 763–773 10.1016/j.neuropsychologia.2009.10.029 19913041

[B13] Boring E. G. (1957). A History of Experimental Psychology. New York, NY: Prenctice Hall

[B14] Borst G. Kosslyn S. M. (2008). Visual mental imagery and visual perception: structural equivalence revealed by scanning processes. Mem. Cognit. 36, 849–862 18604966 10.3758/mc.36.4.849

[B15] Brown S. Martinez M. J. Parsons L. M. (2005). The neural basis of human dance. Cereb. Cortex 16, 1157–1167 10.1093/cercor/bhj057 16221923

[B16] Casasanto D. (2011). Different bodies, different minds: the body specificity of language and thought. Curr. Dir. Psychol. Sci. 20, 378–383

[B17] Casby A. Moran A. (1998). Exploring mental imagery in swimmers: a single-case study design. Ir. J. Psychol. 19, 525–531

[B19] Collet C. Guillot A. Lebon F. MacIntyre T. Moran A. (2011). Measuring motor imagery: combining psychometric, qualitative, chronometric, and psychophysiological techniques. Exerc. Sport Sci. Rev. 39, 89–92 10.1097/JES.0b013e31820ac5e0 21206282

[B20] Confalonieri L. Pagnoni G. Barsalou L. W. Rajendra J. Eickhoff S. B. Butler A. J. (2012). Brain activation in primary motor and somatosensory cortices during motor imagery correlates with motor imagery ability in stroke patients. ISRN Neurol. 2012, 1–17 10.5402/2012/613595 23378930 PMC3544280

[B21] Cornoldi C. De Beni R. (2012). Foreword, in Essays in Honour of Michel Denis: From Mental Imagery to Spatial Cognition and Language, eds Gyselinck V. Pazzaglia F. (Hove, East Sussex: Psychology Press), ix–x

[B22] Cross E. S. (2010). Building a dance in the human brain: Insights from expert and novice dancers, in The Neurocognition of Dance: Mind, Movement and Motor Skills eds Bläsing B. Puttke M. Schack T. (Hove, East Sussex: Psychology Press), 177–202

[B23] Davidson-Kelly K. Hong S. Dhinakaran J. Joseph M. Gray C. (2011). An fMRI study of expert musical imagery: to what extent do imagined and executed performance share the same neural substrate, in Proceedings of the International Symposium on Performance Science 2011, eds Williamon A. Edwards D. Bartel L. (Utrecht: European Association of Conservatoires (AEC), Toronto), 613–617

[B24] Decety J. Jeannerod M. Prablanc C. (1989). The timing of mentally represented actions. Behav. Brain Res. 34, 35–42 2765170 10.1016/s0166-4328(89)80088-9

[B25] Denis M. (2012). Decades of imagery. Reminisences of a shared journey, in Essays in honour of Michel Denis: From Mental Imagery to Spatial Cognition and Language, eds Gyselinck V. Pazzaglia F. (Hove, East Sussex: Psychology Press), 203–254

[B26] Denis M. Carfantan M. (1985). People's knowledge about images. Cognition 20, 49–60 4017520 10.1016/0010-0277(85)90004-6

[B27] Djordjevic J. Zatorre R. J. Petrides M. Boyle J. A. Jones-Gotman M. (2005). Functional neuroimaging of odor imagery. Neuroimage 24, 791–801 10.1016/j.neuroimage.2004.09.035 15652314

[B28] Driskell J. Copper C. Moran A. (1994). Does mental practice enhance performance? A meta-analysis. J. Appl. Psychol. 79, 481–492

[B29] Dror I. Kosslyn S. M. Waag W. (1993). Visual-spatial abilities of pilots. J. Appl. Psychol. 78, 763–773 17760289

[B30] Ericsson K. A. (2009). Development of Professional Expertise: Toward Measurement of Expert Performance and Design of Optimal Learning Environments. Cambridge, MA: Cambridge University Press

[B31] Ericsson K. A. Krampe R. T. Tesch-Römer C. (1993). The role of deliberate practice in the acquisition of expert performance. Psychol. Rev. 100, 363–406

[B32] Ericsson K. A. Smith J. (1991). Prospects and limits in the empirical study of expertise: an introduction, in Toward a General Theory of Expertise: Prospects and Limits, eds Ericsson K. A. Smith J. (Cambridge: Cambridge University Press), 1–38

[B33] Farah M. J. (2000a). The neural basis of mental imagery, in The Cognitive Neurosciences, ed Gazzaniga M. S. (Cambridge, MA: MIT Press), 965–974

[B34] Farah M. J. (2000b). Interview with Martha Farah. J. Cogn. Neurosci. 12, 360–363 10771419 10.1162/089892900562057

[B35] Fourkas A. D. Bonavolonta V. Avenanta A. Aglioti S. M. (2008). Kinesthetic imagery and tool-specific modulation of corticospinal representations in expert tennis players. Cereb. Cortex 18, 2382–2390 10.1093/cercor/bhn005 18296436

[B36] Foxe J. J. Molholm S. (2009). Ten years at the Multisensory Forum: musings on the evolution of a field. Brain Topogr. 21, 149–154 10.1007/s10548-009-0102-9 19452270

[B37] Gabbard P. Cacola P. Bobbio T. (2012). The ability to mentally represent action is associated with low motor imagery ability in children: a preliminary investigation. Child Care Health Dev. 38, 390–393 10.1111/j.1365-2214.2011.01257.x 21651610

[B38] Gallese V. Sinigaglia C. (2012). What is so special about embodied simulation? Trends Cogn. Neurosci. 15, 512–519 10.1016/j.tics.2011.09.003 21983148

[B39] Galton F. (1883). Enquiries into Human Faculty. London: Dent

[B41] Guillot A. Collet C. (2005). Duration of mentally simulated movement: a review. J. Mot. Behav. 37, 10–20 10.3200/JMBR.37.1.10-20 15642689

[B42] Guillot A. Collet C. (2008). Construction of the motor imagery integrative model in sport: a review and theoretical investigation of motor imagery use. Int. Rev. Sport Exerc. Psychol. 1, 31–44

[B43] Guillot A. Collet C. (2010). The Neurophysiological Foundations of Mental and Motor Imagery. Oxford: Oxford University Press

[B45] Guillot A. Di Rienzo F. MacIntyre T. Moran A. Christian C. (2012a). Imagining is not doing but involves specific motor commands: a review of experimental data related to motor inhibition. Front. Hum. Neurosci. 6:247 10.3389/fnhum.2012.00247 22973214 PMC3433680

[B44] Guillot A. Hoyek N. Louis M. Collet C. (2012b). Learning by thinking: review and future directions for the timing of imagined movements. Int. Rev. Sport Exerc. Psychol. 5, 3–22

[B47a] Guillot A. Moschberger K. Collet C. (2013). Coupling movement with imagery as a new perspective for motor imagery practice. Behav. Brain Funct. 9, 1–17 10.1186/1744-9081-9-8 23425312 PMC3599464

[B47] Holmes P. S. Collins D. J. (2001). The PETTLEP approach to motor imagery: a functionalequivalence model for sport psychologists. J. Appl. Sport Psychol. 13, 60–83

[B48] Hubbard T. L. (2010). Auditory imagery: empirical findings. Psychol. Bull. 136, 302–329 10.1037/a0018436 20192565

[B49a] Ietswaart M. Johnston M. Dijkerman H. C. Joice S. Scott C. L. MacWalter R. S. (2011). Mental practice with motor imagery in stroke recovery: randomized controlled trial of efficacy. Brain 134, 1373–1386 10.1093/brain/awr077 21515905 PMC3097892

[B49] Ionta S. Perruchoud D. Draganski B. Blanke O. (2012). Body context and posture affect mental imagery of hands. PLoS ONE 7:e34382 10.1371/journal.pone.0034382 22479618 PMC3316677

[B50] Isaac A. R. Marks D. F. (1994). Individual differences in mental imagery experience: developmental changes and specialisation. Br. J. Psychol. 85, 479–500 7812670 10.1111/j.2044-8295.1994.tb02536.x

[B51] James W. (1890). Principles of Psychology. New York, NY: Holt, Rinehart and Winston

[B53] Jeannerod M. (1994). The representing brain: neural correlates of motor intention and imagery. Behav. Brain Sci. 17, 187–202

[B54] Jeannerod M. (2001). Neural simulation of action: a unifying mechanism for motor cognition. Neuroimage 14, S103–S109 10.1006/nimg.2001.0832 11373140

[B55] Jeannerod M. (2006a). Motor Cognition. New York, NY: Oxford University Press

[B56] Jeannerod M. (2006b). The origin of voluntary action. History of a physiological concept. C. R. Biol. 329, 354–362 10.1016/j.crvi.2006.03.017 16731493

[B57] Kosslyn S. M. (1980). Image and Mind. Cambridge, MA: Harvard University Press

[B58] Kosslyn S. M. (1994). Image and Brain. Cambridge, MA: MIT Press

[B59] Kosslyn S. M. (2010). Multimodal images in the brain, in The Neurophysiological Foundations of Mental and Motor Imagery, eds Guillot A. Collet C. (New York, NY: Oxford University Press), 3–16

[B57a] Kosslyn S. M. Ball T. M. Reiser B. J. (1978). Visual images preserve metric spatial information: evidence from studies of image scanning. J. Exp. Psychol. Hum. 4, 47–60 627850 10.1037//0096-1523.4.1.47

[B60] Kosslyn S. M. Behrmann M. Jeannerod M. (1995). The cognitive neuroscience of mental imagery. Neuropsychologia 33, 1335–1344 10.1016/0028-3932(95)00067-D 8584172

[B61] Kosslyn S. M. Cacioppo J. T. Davidson R. J. Hugdahl K. Lovallo W. R. Spiegel D. (2002). Bridging psychology and biology: the analysis of individuals in groups. Am. Psychol. 57, 341–351 12025764

[B62] Kosslyn S. M. Digirolamo G. J. Thompson W. L. Alpert N. M. (1998). Mental rotation of objects versus hands: neural mechanisms revealed by positron emission tomography. Psychophysiology 35, 151–161 9529941

[B63] Kosslyn S. M. Ganis G. Thompson W. L. (2001a). Neural foundations of imagery. Nat. Rev. Neurosci. 2, 635–642 10.1038/35090055 11533731

[B64] Kosslyn S. M. Thompson W. L. Wraga M. Alpert N. M. (2001b). Imagining rotation by endogenous and exogenous forces: Distinct neural mechanisms for different strategies. Neuroreport 12, 2519–2525 11496141 10.1097/00001756-200108080-00046

[B65] Kosslyn S. M. Koenig O. (1992). Wet Mind. The New Cognitive Neuroscience. New York/Toronto: The Free Press

[B66] Kosslyn S. M. Moulton S. T. (2009). Mental imagery and implicit memory, in Handbook of Imagination and Mental Simulation, eds Markman K. D. Klein W. M. P. Suhr J. A. (New York, NY: Psychology Press), 35–52

[B67] Kosslyn S. M. Pascual-Leone A. Felician O. Camposano S. Keenan J. P. Thompson W. L. (1999). The role of area 17 in visual imagery: convergent evidence from PET and rTMS. Science 284, 167–170 10.1126/science.284.5411.167 10102821

[B68] Kosslyn S. M. Seger C. Pani J. Hillger L. A. (1990). When is imagery used in everyday life? A diary study. J. Ment. Imagery 14, 131–152

[B69] Kosslyn S. M. Shephard J. M. Thompson W. L. (2007). Spatial processing during mental imagery: a neurofunctional theory, in Spatial Processing in Navigation, Imagery, and Perception, eds Mast F. Jancke L. (New York, NY: Springer), 1–16

[B70] Kosslyn S. M. Thompson W. L. Ganis G. (2006). The Case for Mental Imagery. Oxford: Oxford University Press

[B71] Kozhevnikov M. Blajenkova O. (2013). Individual differences in object versus spatial imagery: from neural correlates to real-world applications, in Theoretical and Applied Perspectives on Multisensory Imagery, eds Lacey S. Lawson R. (New York, NY: Springer), 299–318

[B72] Lacey S. Lawson R. (2013). Theoretical and Applied Perspectives on Multisensory Imagery. New York, NY: Springer

[B73] Leone G. Taine M. C. Droulez J. (1993). The influence of long-term practice on mental rotation strategies of 3-D objects. Brain Res. Cogn. Brain Res. 1, 241–255 8003923 10.1016/0926-6410(93)90008-s

[B74] MacIntyre T. Moran A. (2007a). A qualitative investigation of imagery use and meta-imagery processes among elite canoe-slalom competitors. J. Imagery Res. Sport Phys. Act. 2, 1–23

[B75] MacIntyre T. Moran A. (2007b). A qualitative investigation of meta-imagery processes and imagery direction among elite athletes. J. Imagery Res. Sport Phys. Act. 2, 1–20

[B76] MacIntyre T. Moran A. (2010). Meta-imagery processes among elite sports performers, in The Neurophysiological Foundations of Mental and Motor Imagery, eds Guillot A. Collet C. (New York, NY: Oxford University Press), 227–244

[B77] Madan C. R. Singhal A. (2012). Motor imagery and higher level cognition: four hurdles before research can sprint forward. Cogn. Process. 13, 211–229 10.1007/s10339-012-0438-z 22466605

[B78] Malouin F. Richards C. L. (2013). Clinical applications of motor imagery in rehabilitation, in Theoretical and Applied Perspectives on Multisensory Imagery, eds Lacey S. Lawson R. (New York, NY: Springer), 397–420

[B79] Marks D. F. (1973). Visual imagery differences in the recall of pictures. Br. J. Psychol. 64, 17–24 4742442 10.1111/j.2044-8295.1973.tb01322.x

[B80] McAvinue L. P. Robertson I. H. (2007). Measuring motor imagery ability: a review. Eur. J. Cogn. Psychol. 20, 232–251

[B81] McEwan S. E. Hujbregts M. P. J. Ryan J. D. Polatajko H. J. (2009). Cognitive strategy use to enhance motor skill acquisition post-stroke: a critical review. Brain Injury 23, 263–277 10.1080/02699050902788493 19277922

[B82] Meister I. G. Krings T. Foltys H. Boroojerdi B. Muller M. Topper R. (2004). Playing piano in the mind: an fMRI study on music imagery and performance in pianists. Brain Res. Cogn. Brain Res. 19, 219–228 10.1016/j.cogbrainres.2003.12.005 15062860

[B84] Milton J. Solodkin A. Hlustik P. Small S. L. (2007). The mind of expert performance is cool and focused. Neuroimage 35, 804–813 10.1016/j.neuroimage.2007.01.003 17317223

[B85] Moran A. (2002). In the mind's eye. Psychologist 15, 414–415 10.1016/j.conb.2005.03.017 15831399

[B86] Moran A. (2009). Cognitive psychology in sport: progress and prospects. Psychol. Sport Exerc. 10, 420–426

[B87] Moran A. Guillot A. MacIntyre T. Collet C. (2012). Re-imagining motor imagery: building bridges between cognitive neuroscience and sport psychology. Br. J. Psychol. 103, 224–247 10.1111/j.2044-8295.2011.02068.x 22506748

[B88] Moran A. P. (2012). Sport and Exercise Psychology: A Critical Introduction. London: Routledge

[B89] Moran A. P. MacIntyre T. (1998). “There's more to an image than meets the eye”: a qualitative study of kinaesthetic imagery among elite canoe-slalomists. Ir. J. Psychol. 19, 406–423

[B90a] Morris A. Spittle A. (2012). Target article: a default hypothesis of the development of internal and external imagery perspectives. J. Mental Imagery 36, 1–30

[B90] Morris T. Spittle M. Watt A. P. (2005). Imagery in Sport. Champaign, IL: Human Kinetics

[B91a] Munroe K. Giacobbi P. Hall C. Weinberg R. (2000). The four W's of imagery use: where, when, why, and what. Sport Psychol. 14, 119–137 17479579

[B92] Munzert J. Lorey J. Zentgraf J. (2009). Cognitive motor processes: the role of motor imagery in the study of motor representations. Brain Res. Rev. 60, 306–326 10.1016/j.brainresrev.2008.12.024 19167426

[B91] Munzert J. Zentgraf J. (2009). Motor imagery and its implications for understanding the motor system. Prog. Brain Res. 174, 219–229 10.1016/S0079-6123(09)01318-1 19477342

[B93] Neisser U. (1976). Cognition and Reality: Principles and Implications of Cognitive Psychology. New York, NY: WH Freeman

[B94] Neisser U. (1978). Memory: What are the important questions? in Practical Aspects of Memory, eds Gruneburg M. M. Morris P. E. Sykes R. N. (London: Academic Press), 3–24

[B95] Nielsen B. J. Cohen L. G. (2008). The Olympic brain. Does corticospinal plasticity play a role in the acquisition of skills required for high performance sports? J. Physiol. 586, 65–70 10.1113/jphysiol.2007.142661 17717010 PMC2375560

[B96] Nikulin V. V. Hohlefeld F. U. Jacobs A. M. Curio G. (2008). Quasi-movements: a novel motor-cognitive phenomena. Neuropsychologia 46, 727–742 10.1016/j.neuropsychologia.2007.10.008 18035381

[B97] Nitschke J. B. Dixon G. E. Sarinopoulos I. Short S. J. Cohen J. D. Smith E. E. (2006). Altering expectancy dampens neural response to aversive taste in primary taste cortex. Nat. Neurosci. 9, 435–442 10.1038/nn1645 16462735

[B98] Nordin S. M. Cumming J. (2005). Professional dances describe their imagery: where when, what, why, and how. Sport Psychol. 19, 395–416

[B100] Olson C. J. Nyberg L. (2011). Brain simulation of action may be grounded in physical experience. Neurocase 17, 501–505 10.1080/13554794.2010.547504 21506042

[B101] Olsson C. J. Johnsson B. Larsson A. Nyberg L. (2008). Motor representations and practice affect brain systems underlying imagery: an fMRI study of internal imagery in novices and active high jumpers. Open Neuroimag. J. 2, 5–13 10.2174/1874440000802010005 19018312 PMC2577943

[B102] Paivio A. Yuille J. C. Madigan S. A. (1968). Concreteness, imagery, and meaningfulness values for 925 nouns. J. Exp. Psychol. 76, 1–15 5672258 10.1037/h0025327

[B103] Pascual-Leone A. Nguyet D. Cohen L. G. Brasil-Neto J. P. Cammarota A. Hallett M. (1995). Modulation of muscle responses evoked by transcranial magnetic stimulation during the acquisition of new fine motor skills. J. Neurophysiol. 74, 1037–1045 7500130 10.1152/jn.1995.74.3.1037

[B104] Pearson D. G. Deeprose C. Wallace-Hadrill S. M. A. Heyes S. B. Holmes E. A. (2012). Assessing mental imagery in clinical psychology: a review of imagery measures and a guiding framework. Clin. Psychol. Rev. 33, 1–23 10.1016/j.cpr.2012.09.001 23123567 PMC3545187

[B105] Pearson J. Clifford C. W. G. Tong F. (2008). The functional impact of mental imagery on conscious perception. Curr. Biol. 18, 982–986 10.1016/j.cub.2008.05.048 18583132 PMC2519957

[B106] Pearson J. Rademaker R. Tong F. (2011). Evaluating the mind's eye: the metacognition of visual imagery. Psychol. Sci. 22, 1535–1542 10.1177/0956797611417134 22058106

[B107] Perky C. W. (1910). An experimental study of imagination. Am. J. Psychol. 21, 422–452

[B109] Pylyshyn Z. (2003). Explaining mental imagery: now you see it, now you don't. Trends Cogn. Sci. 7, 111–112 10.1016/S1364-6613(03)00004-4 12639691

[B110] Rademaker R. L. Pearson J. (2012). Training visual imagery: improvements of metacognition, but not imagery strength. Front. Psychol. 3:224 10.3389/fpsyg.2012.00224 22787452 PMC3392841

[B111] Richardson A. (1967a). Mental practice: a review and discussion, Part 1. Res. Q. 38, 95–107 5338007

[B112] Richardson A. (1967b). Mental practice: a review and discussion, Part II. Res. Q. 38, 263–273 5338007

[B114] Roekelein J. E. (2004). Imagery and Psychology: A Reference Guide. Westport, CT: Praeger

[B115] Rosenbaum D. A. (2005). The Cinderella of psychology: the neglect of motor control in the science of mental life and behavior. Am. Psychol. 60, 308–317 10.1037/0003-066X.60.4.308 15943523

[B116] Ross J. S. Thach J. Ruggieri P. M. Lieber M. Lapresto E. (2003). The mind's eye: functional MR imaging evaluation of golf motor imagery. Am. J. Neuroradiol. 24, 1036–1044 12812924 PMC8149015

[B117] Saarliluoma P. Karlsson H. Lyytinen H. Teras M. Geisler F. (2004). Visuospatial representations used by chess experts: a preliminary study. Eur. J. Cogn. Psychol. 16, 753–766

[B118] Sarter M. Berntson G. G. Cacioppo J. T. (1996). Brain imaging and cognitive neuroscience: toward strong inference in attributing function to structure. Am. Psychol. 51, 13–21 8585670 10.1037//0003-066x.51.1.13

[B119] Schmuckler M. A. (2001). What is ecological validity? A dimensional analysis. Infancy 2, 419–436 10.1207/S15327078IN0204_02 33451194

[B120] Senior C. T. Russell T. Gazzaniga M. S. (2006). Methods in Mind. Cambridge, MA: MIT Press

[B121] Sevdalis N. Moran A. Arora S. (2013). Mental imagery and mental practice applications in surgery, in Multisensory Imagery, eds Lacey S. Lawson R. (New York, NY: Springer), 343–363

[B122] Shepard R. N. Metzler J. (1971). Mental rotation of three dimensional objects. Science 171, 701–703 10.1126/science.171.3972.701 5540314

[B123] Slotnick S. D. Thompson W. L. Kosslyn S. M. (2005). Visual mental imagery induces retinotopically organized activation of early visual areas. Cereb. Cortex 15, 1570–1583 10.1093/cercor/bhi035 15689519

[B124] Smith E. E. Kosslyn S. M. (2007). Motor cognition and mental simulation, in Cognitive Psychology: Mind and Brain, eds Smith E. E. Kosslyn S. M. (Upper Saddle River, NJ: Prentice Hall), 451–481

[B125] Smyth M. M. Waller A. (1998). Movement imagery in rock climbing: patterns of interference from visual, spatial and kinaesthetic secondary tasks. Appl. Cogn. Psychol. 12, 145–157

[B126] Steiger J. H. Yuille J. C. (1983). Long term memory and mental rotation. Can. J. Psychol. 37, 367–389 6640445 10.1037/h0080740

[B127] Stewart L. Walsh V. (2006). Transcranial magnetic stimulation in cognition, in Methods in Mind, eds Senior C. Russell T. Gazzaniga M. S. (Cambridge, MA: MIT Press), 1–26

[B128] Tartaglia E. M. Bamert L. Mast F. W. Herzog M. H. (2009). Human perceptual learning by mental imagery. Curr. Biol. 19, 2081–2085 10.1016/j.cub.2009.10.060 19962313

[B129] ter Horst A. C. Cole J. van Lier R. Steenbergen B. (2012). The effect of chronic deafferentation on mental imagery: a case study. PLoS ONE 7:e42742 10.1371/journal.pone.0042742 22880095 PMC3413668

[B130] Van den Auweele Y. Depreeuw E. Rzewnicki R. Ballon F. (1993). Elite performance and personality: from description and prediction to diagnosis and intervention, in Handbook of Research on Sport Psychology, eds Singer R. N. Murphey M. Tennant L. K. (New York, NY: Macmillan), 257–289

[B131] Vandell R. A. Davis R. A. Clugston H. A. (1943). The function of mental practice in the acquisition of motor skills. J. Gen. Psychol. 29, 243–250

[B132] Wakefield C. J. Smith D. Moran A. Holmes P. (2013). Functional equivalence or behavioural matching? A critical reflection on 15 years of research using the PETTLEP model of motor imagery. Int. Rev. Sport Exerc. Psychol. 6, 105–121

[B133] Walsh V. Cowey A. (2000). TMS and cognitive neuroscience. Nat. Rev. Neurosci. 1, 73–79 10.1038/35036239 11252771

[B134] Weinberg R. S. (2008). Does imagery work? Effects on performance and mental skills. J. Imagery Res. Sport Phys. Act. 3, 1–21

[B135] Welfringer A. Leifert-Fiebach G. Babinsky R. Brandt T. (2011). Visuomotor imagery as a new tool in the rehabilitation of neglect: a randomised controlled study of feasibility and efficacy. Disabil. Rehabil. 33, 2033–2043 10.3109/09638288.2011.556208 21348577

[B136] Williams S. E. Cumming J. (2011). Measuring athlete imagery ability: the sport imagery ability questionnaire. J. Sport Exerc. Psychol. 33, 416–440 21659671 10.1123/jsep.33.3.416

[B137] Wraga M. Kosslyn S. M. (2002). Imagery, in Encyclopedia of Cognitive Science, Vol. 2 (London: Nature Publishing Group), 466–470

[B138] Yarrow K. Brown P. Krakauer J. W. (2009). Inside the brain of an elite athlete: the neural processes that support high achievement in sports. Nat. Rev. Neurosci. 10, 585–596 10.1038/nrn2672 19571792

